# 

**Published:** 1840-11

**Authors:** 


### WESTERN JOURNAL

OF

### MEDICINE AND SURGERY:

EDITED BY

DANIEL DRAKE, M. D.

AND

LUNSFORD P. YANDELL, M. D.

PROFESSORS IN THE LOUISVILLE MEDICAL INSTITUTE.

NO. XI.—NOVEMBER, 1840.

VOL. II.—NO. V.

LOUISVILLE, KY.

PUBLISHED MONTHLY, BY PRENTICE & WEISSINGER,

PROPRIETORS AND PRINTERS.

1840.

*This Number contains* 4 *sheets—Postage under* 100 *miles* 6 *cents, over* 100 *miles* 10 *cents.*

